# Chronic Central Serous Chorioretinopathy in a Patient with Pigment Dispersion Syndrome: A Possible Correlation

**DOI:** 10.1155/2017/5857041

**Published:** 2017-10-01

**Authors:** Dimitrios Kourkoutas, George Tsakonas, Aristotelis Karamaounas, Nikolaos Karamaounas

**Affiliations:** Department of Ophthalmology, 401 Army General Hospital of Athens, Athens, Greece

## Abstract

Chronic central serous chorioretinopathy (CSCR) is a progressive chorioretinopathy with widespread atrophic RPE abnormalities and serous retinal detachments (SRDs) present for 6 months or longer. We report a case of CSCR in a 38-year-old patient with Pigment Dispersion Syndrome (PDS). In the presented case of CSCR, the chronic course of the disease may in part be associated with an underlying generalized degenerative dysfunction of the pigmented cells of the eye on grounds of PDS. We suggest that a chronic course of disease may be suspected in the setting of CSCR with concurrent RPE pathology, such as what is found in PDS.

## 1. Introduction

Pigment dispersion syndrome (PDS) [1] is characterized by the classic diagnostic triad of corneal endothelial pigmentation (Krukenberg spindle), radial, mid-peripheral iris transillumination defects, and dense homogeneous trabecular meshwork pigmentation. In PDS, the iris anatomy results in disruption of the iris pigment epithelium (IPE) and dispersion of iris pigment in the anterior segment of the eye. In addition, electrophysiological studies have suggested that the functional integrity of the RPE/photoreceptor complex is affected in eyes with PDS and pigmentary glaucoma [2–4]. The prevalence of PDS has been described to be between 2% and 4% in a white population between the ages of 20 and 40 years [5].

Central serous chorioretinopathy (CSCR) [6] is defined as an idiopathic condition that leads to retinal pigment epithelium (RPE) detachments and serous detachments of the neurosensory retina at the macula. CSCR has been estimated as the fourth most frequent nonsurgical retinopathy after age-related macular degeneration (AMD), diabetic retinopathy, and branch retinal vein occlusion [6]. More specifically, chronic CSCR represents approximately 5% of CSCR cases [7], while follow-up studies indicate a progression from acute to chronic CSCR in approximately 16% of cases [8].

CSCR can be differentiated into three types: acute, recurrent, and chronic [6, 9]. In acute CSCR, RPE changes are mostly localized while recurrent CSCR refers to a new acute CSCR episode following a previous completely resolved episode. Acute CSCR cases with serous retinal detachments (SRDs) present for 6 months or longer after onset of symptoms are characterized as chronic CSCR [10, 11] and are associated with more severe and widespread RPE disease with or without SRD, frequently referred to as diffuse retinal pigment epitheliopathy (DRPE). In chronic CSCR, abnormal choroidal circulation and extensive dysfunction and loss of RPE result in longstanding subretinal fluid, photoreceptor death, secondary choroidal neovascularization (CNV), and permanent visual loss. Even though the current understanding of the pathogenesis of CSCR supports the role of the choroid, RPE dysfunction seems to play a significant role especially in the pathogenesis of chronic cases.

We report, for the first time, a case of concurrent chronic CSCR and PDS.

## 2. Case Presentation

**Figure 1:**
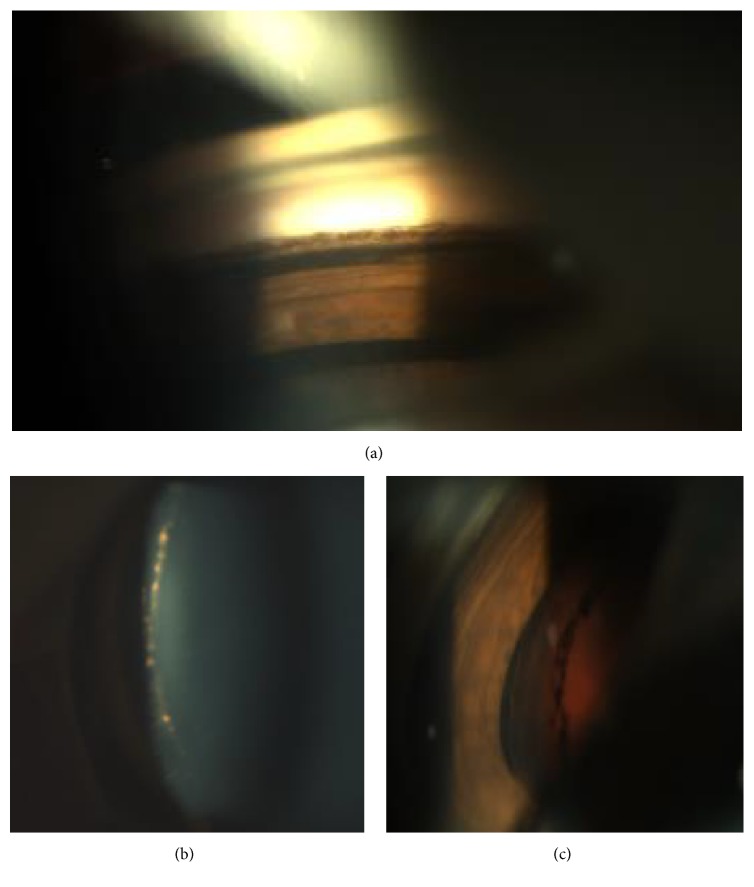
(a) Increased trabecular pigmentation (b) (c) Zentmayer ring. Pigment accumulated at the zonular attachments to the lens.

**Figure 2:**
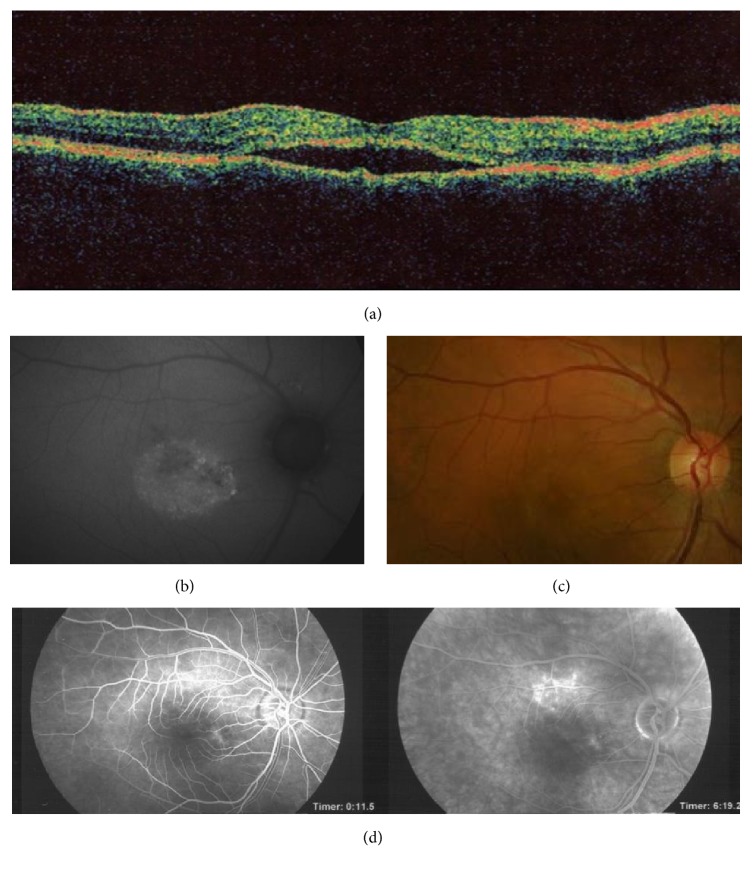
Optical coherence tomography (a) is showing pigment epithelial detachment of the neurosensory retina. Fundus photography (b, c) and fluorescein angiography (d) in the right eye of a 38-year-old male patient. The patient had suffered, to variable degrees, from painless blurred vision, dyschromatopsia, and micropsia for more than 2 years. Fundus abnormalities included an irregular pattern of hypopigmentation of the retinal pigment epithelium, amorphous subretinal deposits, and a shallow serous detachment involving large parts of the posterior pole. These findings are highly suggestive of chronic central serous chorioretinopathy.

A 38-year-old Army Officer presented for follow-up after a resolving episode of CSCR in his right eye. The patient had suffered, to variable degrees, from painless blurred vision, dyschromatopsia, and micropsia for more than 2 years. His ocular history included PDS and recurrent CSCR. He denied smoking or use of systemic steroids. He was otherwise healthy with no family history of glaucoma. On examination, best corrected visual acuity was 0.00 ETDRS logMAR OU. Intraocular pressures were normal in both eyes (<21 mmHg). With regard to PDS, a significant asymmetry was noted with prominent features in the right eye including dense Krukenberg spindle, iris transillumination defects, heavily pigmented trabecula, wide open angle, and Zentmayer ring (Figure 1). Fundoscopy revealed right inferior lattice degeneration and healthy optic discs. Small parafoveal areas of hypopigmented retinal pigment epithelium with associated residual subretinal fluid and accompanying drusen were illustrated on colour fundus photographs, fluorescein angiography (FFA), and OCT (Figure 2). These findings were highly suggestive of chronic CSCR. Review of previous images, within the last 24 months, showed 4 recurrent episodes of serous detachment of the neurosensory retina compatible with right eye chronic CSCR.

## 3. Discussion

Several aspects have been implicated in the pathogenesis of CSCR such as increased permeability of the choriocapillaris, a failing Bruch's membrane, and a defect in the diffusion-barrier function of the RPE. The anatomic predilection of the disease for the macular area is possibly due to the special histological features of the retina and RPE as well as the particular pattern of the submacular choroidal vascular bed with a densely organized meshwork compared to peripheral choroid [12]. Regardless of the primary cause or the initiating event, the source of the subretinal fluid in CSCR is the choriocapillaris-Bruch's membrane-RPE layer. It has been therefore suggested that there could be a combination of increased fluid leakage from the choriocapillaris and impaired RPE function [13].

The role of the RPE is still not well understood in the pathogenesis of CSCR [6, 14]. Although it remains uncertain whether epitheliopathy and choroidopathy both share common pathogenic mechanisms, RPE integrity plays an important role against the high choroidal hydrostatic pressure, even in mild or asymptomatic cases [15, 16]. Of note, morphologic alterations of the macular RPE are present, on SD-OCT 3D single-layer RPE analysis, in nearly all (94% of eyes) asymptomatic contralateral eyes of CSCR patients [14].

The distinction between acute and chronic CSCR is based on the duration of the SRD and on the extension of RPE involvement. The chronic course of the disease is associated with diffuse epitheliopathy (DRPE) which is clinically detected as pigment epithelium detachments (PEDs), RPE rips, widespread areas of RPE atrophy, and areas of RPE hypertrophy [17, 18].

Furthermore, recent studies have drawn attention to peripheral retinal pathology and rhegmatogenous retinal detachment (RRD) which were reported to be more common in patients with CSCR compared to healthy subjects [19]. In addition, Oztas et al. [20] reported that peripheral retinal degeneration and lattice degeneration were more common in the chronic CSCR group than in the acute CSCR group. These findings may reflect a generalized RPE abnormality especially in chronic CSCR patients.

Associations of PDS with lattice degeneration, rhegmatogenous retinal detachment, and pigmentary retinal dystrophy have been reported in the literature [21, 22]. PDS is associated with a 6% to 7% incidence of retinal detachment. Ritch has suggested that the association with retinal lattice degeneration, retinal tears, and retinal detachment could be explained by a genetic abnormality affecting the middle third of the eye during the third trimester [22]. Both the iris pigmented epithelial (IPE) and the RPE cells are affected, as they share a common embryological origin, namely, the external layer of the optic vesicle. A few clinical observations have also reported a widespread degeneration of the RPE associated with pigmentary glaucoma and PDS [23–25]. Previous findings of generalized RPE dysfunction in eyes with PDS or pigmentary glaucoma (PG) as determined by electrooculography are very interesting [4].

To our knowledge, this is the first reported case of a patient with concurrent chronic CSCR and PDS. In the presented case of CSCR, the chronic course of the disease may in part be associated with an underlying generalized degenerative dysfunction of the pigmented cells of the eye on grounds of PDS. Our case supports the theory that impaired RPE function in the setting of choroidal hyperpermeability results in chronic subretinal fluid.

Chronic CSCR is a progressive chorioretinopathy which may not share the same pathophysiology as that of acute CSCR. More importantly, chronic CSCR has a significant impact on vision-related quality of life as many chronic CSCR patients are experiencing significant vision loss [26]. Therefore, identification of factors which are possibly associated with chronicity may aid in improving the management of these patients. The goals of improved disease management would be to anticipate disease natural history and prognosis, allow patient counseling, and make timely and suitable treatment decisions.

In conclusion, this case report highlights the possible intriguing association between chronic CSCR and PDS and sheds some light on the putative pathogenetic mechanisms (namely, RPE abnormality), although this association could be fortuitous. Further studies are necessary to determine the pathophysiological significance of RPE dysfunction in chronic CSCR. We suggest that a chronic course of disease may be suspected in the setting of CSCR with concurrent RPE pathology, such as what is found in PDS.

## References

[1] Niyadurupola N., Broadway D. C. (2008). Pigment dispersion syndrome and pigmentary glaucoma - A major review. *Clinical and Experimental Ophthalmology*.

[2] Scuderi G. L., Ricci F., Nucci C., Galasso M. J., Cerulli L. (1998). Electro-oculography in pigment dispersion syndrome. *Ophthalmic Research*.

[3] Demailly P., Plane C., Limon S., Luton J. P.

[4] Greenstein V. C., Seiple W., Liebmann J., Ritch R. (2001). Retinal pigment epithelial dysfunction in patients with pigment dispersion syndrome: Implications for the theory of pathogenesis. *Archives of Ophthalmology*.

[5] Ritch R., Steinberger D., Liebmann J. M. (1993). Prevalence of pigment dispersion syndrome in a population undergoing glaucoma screening. *American Journal of Ophthalmology*.

[6] Wang M., Munch I. C., Hasler P. W., Prünte C., Larsen M. (2008). Central serous chorioretinopathy. *Acta Ophthalmologica*.

[7] Spaide R. F., Campeas L., Haas A. (1996). Central serous chorioretinopathy in younger and older adults. *Ophthalmology*.

[8] Castro-Correia J., Coutinho M. F., Rosas V., Maia J. (1992). Long-term follow-up of central serous retinopathy in 150 patients. *Documenta Ophthalmologica*.

[9] Ross A., Ross A. H., Mohamed Q. (2011). Review and update of central serous chorioretinopathy. *Current Opinion in Ophthalmology*.

[10] Yannuzzi L. A. (2010). Central Serous Chorioretinopathy: A Personal Perspective. *American Journal of Ophthalmology*.

[11] Yannuzzi L. A., Slakter J. S., Kaufman S. R., Gupta K. (1992). Laser treatment of diffuse retinal pigment epitheliopathy.. *European journal of ophthalmology*.

[12] Hayreh S. S. (1975). Segmental nature of the choroidal vasculature. *British Journal of Ophthalmology*.

[13] S. J. Ryan (2001). Central serous chorioretinopathy. *Retina*.

[14] Gupta P., Gupta V., Dogra M. R., Singh R., Gupta A. (2010). Morphological changes in the retinal pigment epithelium on spectral-domain OCT in the unaffected eyes with idiopathic central serous chorioretinopathy. *International Ophthalmology*.

[15] Ferrara D., Mohler K. J., Waheed N. (2014). En face enhanced-depth swept-source optical coherence tomography features of chronic central serous chorioretinopathy. *Ophthalmology*.

[16] Fujimoto H., Gomi F., Wakabayashi T., Sawa M., Tsujikawa M., Tano Y. (2008). Morphologic Changes in Acute Central Serous Chorioretinopathy Evaluated by Fourier-Domain Optical Coherence Tomography. *Ophthalmology*.

[17] Lim Z., Wong D. (2008). Retinal pigment epithelial rip associated with idiopathic central serous chorioretinopathy [2]. *Eye*.

[18] Yang L., Jonas J. B., Wei W. (2013). Optical coherence tomography-assisted enhanced depth imaging of central serous chorioretinopathy. *Investigative Ophthalmology & Visual Science*.

[19] Chang Y.-S., Chang C., Weng S.-F., Wang J.-J., Jan R.-L. (2016). Risk of rhegmatogenous retinal detachment with central serous chorioretinopathy. *Retina*.

[20] Oztas Z., Akkin C., Ismayilova N., Nalcaci S., Afrashi F. (2017). The importance of the peripheral retina in patients with central serous chorioretinopathy. *Retina*.

[21] Weseley P., Liebmann J., Walsh J. B., Ritch R. (1992). Lattice degeneration of the retina and the pigment dispersion syndrome. *American Journal of Ophthalmology*.

[22] Mudumbai R., Liebmann J., Ritch R. (2000). Combined exfoliation and pigment dispersion: an overlap syndrome. Trans Am Ophthalmol Soc 1999;99:297–314.. *American Journal of Ophthalmology*.

[23] Chew E. Y., Deutman A. F. (1983). Pigment dispersion syndrome and pigmented pattern dystrophy of retinal pigment epithelium. *British Journal of Ophthalmology*.

[24] Kadayifcilar S., Tatlipinar S., Eldem B., Irkec M. (2000). Pigment dispersion syndrome and butterfly-shaped pattern dystrophy of the retinal pigment epithelium [6]. *Eye*.

[25] Piccolino F. C., Calabria G., Polizzi A., Fioretto M. (1989). Pigmentary retinal dystrophy associated with pigmentary glaucoma. *Graefe's Archive for Clinical and Experimental Ophthalmology*.

[26] Breukink M. B., Dingemans A. J. M., Den Hollander A. I. (2017). Chronic central serous chorioretinopathy: Long-term follow-up and vision-related quality of life. *Clinical Ophthalmology*.

